# Frequency, Risk Factors and Survival Associated with an Intrasubsegmental Recurrence after Radiofrequency Ablation for Hepatocellular Carcinoma

**DOI:** 10.1371/journal.pone.0059040

**Published:** 2013-04-12

**Authors:** Ryosuke Tateishi, Shuichiro Shiina, Masaaki Akahane, Jiro Sato, Yuji Kondo, Ryota Masuzaki, Hayato Nakagawa, Yoshinari Asaoka, Tadashi Goto, Kuni Otomo, Masao Omata, Haruhiko Yoshida, Kazuhiko Koike

**Affiliations:** 1 Department of Gastroenterology, Graduate School of Medicine, The University of Tokyo, Tokyo, Japan; 2 Department of Radiology, Graduate School of Medicine, The University of Tokyo, Tokyo, Japan; 3 Yamanashi Prefectural Hospital Organization, Tokyo, Japan; Icahn School of Medicine at Mount Sinai, United States of America

## Abstract

**Background:**

In the treatment of hepatocellular carcinoma (HCC), hepatic resection has the advantage over radiofrequency ablation (RFA) in terms of systematic removal of a hepatic segment.

**Methods:**

We enrolled 303 consecutive patients of a single naïve HCC that had been treated by RFA at The University of Tokyo Hospital from 1999 to 2004. Recurrence was categorized as either intra- or extra-subsegmental as according to the Couinaud's segment of the original nodule. To assess the relationship between the subsegments of the original and recurrent nodules, we calculated the kappa coefficient. We assessed the risk factors for intra- and extra-subsegmental recurrence independently using univariate and multivariate Cox proportional hazard regression. We also assessed the impact of the mode of recurrence on the survival outcome.

**Results:**

During the follow-up period, 201 patients in our cohort showed tumor recurrence distributed in a total of 340 subsegments. Recurrence was categorized as exclusively intra-subsegmental, exclusively extra-subsegmental, and simultaneously intra- and extra-subsegmental in 40 (20%), 110 (55%), and 51 (25%) patients, respectively. The kappa coefficient was measured at 0.135 (95% CI, 0.079–0.190; P<0.001). Multivariate analysis revealed that of the tumor size, AFP value and platelet count were all risk factors for both intra- and extra-subsegmental recurrence. Of the patients in whom recurrent HCC was found to be exclusively intra-subsegmental, extra-subsegmental, and simultaneously intra- and extra-subsegmental, 37 (92.5%), 99 (90.8%) and 42 (82.3%), respectively, were treated using RFA. The survival outcomes after recurrence were similar between patients with an exclusively intra- or extra-subsegmental recurrence.

**Conclusions:**

The effectiveness of systematic subsegmentectomy may be limited in the patients with both HCC and chronic liver disease who frequently undergo multi-focal tumor recurrence.

## Introduction

Hepatic resection is regarded as the most appropriate first-line treatment for patients with solitary hepatocellular carcinoma (HCC) who are non-cirrhotic or cirrhotic without portal hypertension [1]. Hepatic resection is also indicated for HCC patients with more advanced cirrhosis in countries like Japan where the option of performing a liver transplantation is limited by the scarcity of cadaveric donor organs [2]. As a surgical procedure, anatomical resection, which is the systematic removal of a hepatic segment containing tumor tissue, is considered to be preferable based on the concept that tumor cells disseminate through the portal vein [3]–[8].

Percutaneous tumor ablation methods, such as ethanol injection and microwave coagulation, have played an important role as nonsurgical treatments that can achieve high local cure rates without reducing background liver function [9]–[12]. Radiofrequency ablation (RFA) is currently considered to be the most effective first-line percutaneous ablation protocol because of its greater efficacy in terms of local cure compared with ethanol injection [13]–[16]. The survival outcomes for patients who achieved a complete response by RFA are comparable to that among patients treated by hepatic resection [17]–[20].

Hepatic resection is supposed to have the advantage over RFA as an effective intervention as it involves the systematic removal of a hepatic segment containing the tumor. Indeed, microscopic satellite nodules, not detected by radiological examination prior to resection, are often observed in the resected specimen [5], [6], [21]. However, this does not necessarily mean that microscopic lesions will have been confined to the resected segment. Indeed, even after anatomical resection, the cumulative recurrence rate at 5 years is as high as 50–70% [6]–[8], and it is not known to what extent anatomical resection can reduce HCC recurrence as compared with RFA.

Whereas RFA can reliably eliminate target nodules together with some of the surrounding tissue, most of the liver parenchyma of the tumor-bearing segment is left unablated. In contrast to anatomical resection, it is possible to observe and analyze intra- and extra-subsegmental recurrence by following up patients after ablation. The aim of our present study was to assess the frequency, risk factors and survival outcomes associated with intra-subsegmental HCC recurrence after RFA in comparison with extra-subsegmental recurrence.

## Patients and Methods

### Patients

This retrospective study was conducted according to the ethical guidelines for epidemiological research designed by the Japanese Ministry of Education, Culture, Sports, Science and Technology and Ministry of Health, Labour, and Welfare. The study design was included in a comprehensive protocol of retrospective study at the Department of Gastroenterology, The University of Tokyo Hospital approved by The University of Tokyo Medical Research Center Ethics Committee (approval number 2058). The following statements were posted at a website (http://gastro.m.u-tokyo.ac.jp/med/0602A.htm) and participants who do not agree to the use of their clinical data can claim deletion of them.

Department of Gastroenterology at The University of Tokyo Hospital contains data from our daily practice for the assessment of short-term (treatment success, immediate adverse events etc.) and long-term (late complications, recurrence etc.) outcomes. Obtained data were stored in an encrypted hard disk separated from outside of the hospital. When reporting analyzed data, we protect the anonymity of participants for the sake of privacy protection. If you do not wish the utilization of your data for the clinical study or have any question on the research content, please do not hesitate to make contact with us.

From 1999 to 2004 a total of 569 patients with HCC underwent RFA as the initial treatment for naïve HCC. Of them, 304 patents had a single nodule. We enrolled 303 of these patients in our current study excluding one patient who could not achieve complete ablation. The inclusion criteria for RFA had been as follows: a total bilirubin level of less than 3 mg/dL, a platelet count of no less than 50×10^3^/mm^3^ and prothrombin activity levels of no less than 50%. Patients with a portal vein tumor thrombosis, refractory ascites, or extrahepatic metastasis were excluded. In general, we performed RFA on patients with three or fewer lesions of 3 cm or less in diameter. However, we also performed ablation on patients beyond these criteria if it was predicted to be clinically effective [22], [23]. We enrolled patients who underwent transcatheter arterial chemoembolization (TACE) prior RFA when the treatments were sequentially performed.

### Diagnosis of HCC

HCC was diagnosed using dynamic computed tomography (CT), with a consideration of hyperattenuation in the arterial phase with washout in the late phase as a definite sign of this disease [24]. Most nodules were also confirmed histopathologically via an ultrasound-guided biopsy.

### Treatment and evaluation

All patients received dynamic CT with a slice thickness of 5 mm within one month prior to ablation for comparison. The interval between the initiation of contrast material infusion and CT image recording was 30 and 120 sec for single detector-row spiral CT (Highspeed Advantage; GE Medical Systems; Milwaukee, WI) and 25, 40 and 120 sec for multidetector-row CT (LightSpeed QX/i GE Medical Systems). The images were presented after axial reconstruction with a slice thickness of 5 mm. RFA was performed on an in-patient basis using a cooled-tip electrode (Covidien, Mansfield, MA) under real-time ultrasound guidance. After 1 to 2 sessions of RFA, dynamic CT was performed to evaluate the treatment efficacy. During the treatment evaluation, we compared the CT findings for early and late phase before ablation and late phase after ablation. A lesion was judged to be completely ablated when the non-enhanced area shown in the late phase of CT post-ablation covered the entire lesion shown in both early and late phase of CT pre-ablation with a safety margin in the surrounding liver parenchyma. We confirmed complete ablation in all slices on which the target nodule was visualized. Patients received additional sessions until complete ablation was confirmed in each nodule. Finally, 303 of the 304 patients enrolled in this study were judged to be completely ablated.

### Assessment of tumor recurrence

The follow-up regimen consisted of blood tests and monitoring of tumor markers in an outpatient setting. Ultrasonography and dynamic CT were also performed every four months. Tumor recurrence was defined as a newly developed lesion on a dynamic CT that showed hyperattenuation in the arterial phase with washout in the late phase. The nomenclature used for the hepatic segments conformed to *The General Rules for the Clinical and Pathological Study of Primary Liver Cancer, Second English Edition* [25]. According to these rules, subsegments 1 to 8 correspond to Couinaud's segment 1 to 8, respectively [26]. All images were independently reviewed by two experienced radiologists (M.A. and J.S.), and a consensus reading was subsequently performed. Recurrence was categorized as either intra- or extra-subsegmental based on the subsegment of the original nodule. When a tumor was located on two or more subsegments, the subsegment where the major part of the tumor was present was adopted. Local tumor progression and neoplastic seeding through a needle tract were considered to be an intrasubsegmental recurrence. Extrahepatic recurrence was defined as extrasubsegmental.

### Treatment of recurrent HCC and Survival Outcomes

When HCC recurrence was identified, patients who met the same criteria used for primary HCC underwent RFA. Survival analysis was performed on a per patient basis. Patients without an indication for RFA due to a multiplicity of recurrent nodules underwent TACE if liver function was categorized as Child-Pugh class B or better. Patients with localized portal tumor invasion were treated by radiotherapy [27]. Patients with tumor invasion to the first branch or main tract of the portal vein were treated with intra-arterial 5-fluorouracil and systemic interferon-α combination therapy [28]. Those with extrahepatic tumor metastasis received systemic chemotherapy if they had well-preserved liver function and a good performance status. Survival time was defined as the interval between the diagnosis of recurrence and the last visit to the outpatient clinic or death up to December 31, 2010. We also analyzed overall survival after the initial RFA. For the analysis start date was set at the day when we perform the first RFA for each patient.

### Statistical analysis

Data were expressed as the mean ± standard deviation (SD) unless otherwise indicated. To assess whether the location of recurrent nodules was independent of the subsegment of the original nodule, we calculated the kappa coefficient and its 95% confidence interval (CI) [29]. A coefficient of 1 indicates that the subsegments of the original and recurrent nodules are identical, whereas a kappa coefficient of 0 indicates that tumor recurrence occurs completely at random. P values were also calculated on the null hypothesis of kappa equal to zero.

To assess the exclusively intra-subsegmental recurrence rate separately from all kinds of recurrence, we used cumulative incidence estimation with competing risk methods [30]. On this analysis, all types of recurrence were categorized as exclusively intra-subsegmental recurrence, exclusively extrasubsegmental recurrence, or simultaneously intra- and extra-subsegmental recurrence. The hazard function of each type of recurrence was estimated using kernel-based methods described by Muller and Wang [31].

We assessed the risk factors for intra- and extra-subsegmental recurrence independently using univariate and multivariate Cox proportional hazard regression. In assessing the risk factor for intra-subsegmental recurrence, patients with exclusively extra-subsegmental recurrence were treated as censored data and vice versa. The following factors were used for these analyses: age, gender, hepatitis B surface antigen positivity, hepatitis C antibody positivity, platelet count, alanine aminotransferase (ALT), tumor size, alpha-fetoprotein (AFP), des-gamma-carboxyprothrombin (DCP) and lens culinaris agglutinin-reactive fraction of AFP (AFP-L3). Factors showing statistical significance as a predictor in univariate analysis were further analyzed using a multivariate Cox proportional hazard regression model with stepwise selection of variables based on the Akaike information criterion (AIC).

We plotted survival curves according to the mode of recurrence (i.e., intra-, extra-subsegmental or both) using the Kaplan-Meier method. Statistical significance among these three groups was assessed using the log-rank test. We also calculated adjusted hazard ratios for survival according to the mode of recurrence using multivariate Cox proportional hazard regression with factors that showed statistical significance in a univariate analysis of survival. Differences with a *P* value of less than 0.05 were considered statistically significant. All statistical analyses were performed with S-Plus Ver. 7 (TIBCO Software Inc., Palo Alto, CA) and R 2.13.0 (http://www.R-project.org).

## Results

### Patient profiles

The enrolled HCC patient cohort in this study consisted of 191 males and 112 females with a mean age of 67.5 years (Table 1). The mean tumor size was 2.5±1.1 cm in diameter. The number of the nodules distributed in subsegments 1 to 8 was 7 (2.3%), 12 (4.0%), 30 (9.9%), 43 (14.2%), 37 (12.2%), 32 (10.6%), 46 (15.2%), and 96 (31.7%), respectively. One hundred one patients underwent TACE before RFA. The median (range) interval between TACE and RFA was 23 (6–71) days.

**Table 1:** Baseline Characteristics of the HCC Patients analyzed in this study (n  =  303).

Variable	n(%)
Age (y)	
mean ± SD	67.5±8.2
Range	44–91
Male sex	191 (63.0)
Viral infection	
HBsAg positive only	28 (9.2)
anti HCVAb positive only	225 (74.3)
Both positive	5 (1.7)
Both negative	35 (11.6)
Alcohol consumption >80 g/day	43 (14.2)
Child-Pugh classification	
Class A	213 (70.3)
Class B	75 (24.8)
Class C	6 (2.0)
Size of tumor (cm)	
mean ± SD	2.5±1.1
≤2.0	106 (35.0)
2.1–3.0	121 (40.0)
>3.0	76 (25.1)
AFP >100 ng/mL	68 (22.4)
DCP >100 mAU/mL	39 (12.9)
AFP-L3 >15%	44 (14.5)

AFP, alpha-fetoprotein; AFP-L3, lens culinaris agglutinin-reactive fraction of AFP; Anti-HCVAb, anti-hepatitis C virus antibody; DCP, des-gamma-carboxy prothrombin; HBsAg, hepatitis B surface antigen.

### HCC recurrence

**Figure 1:**
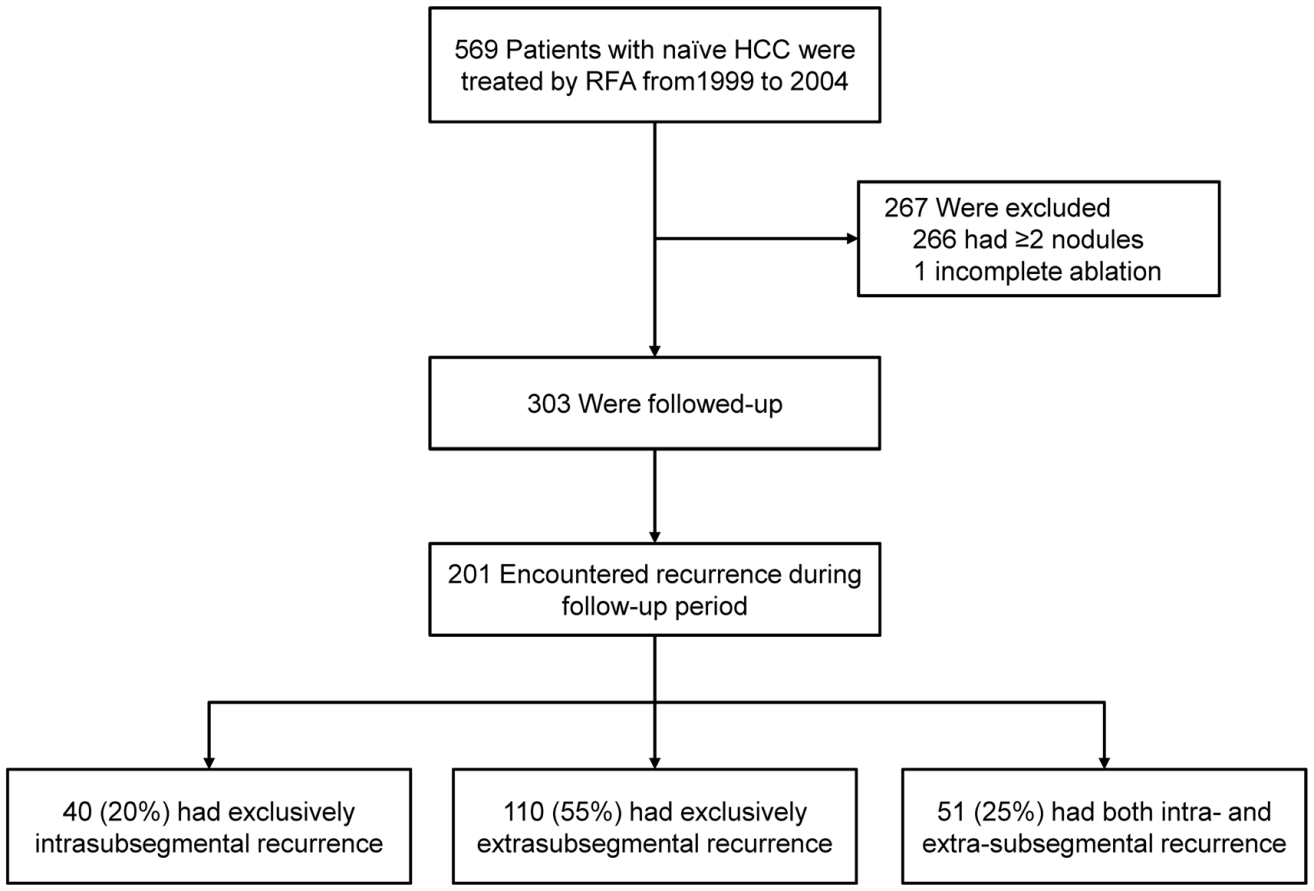
Patient enrollment flow.

**Figure 2:**
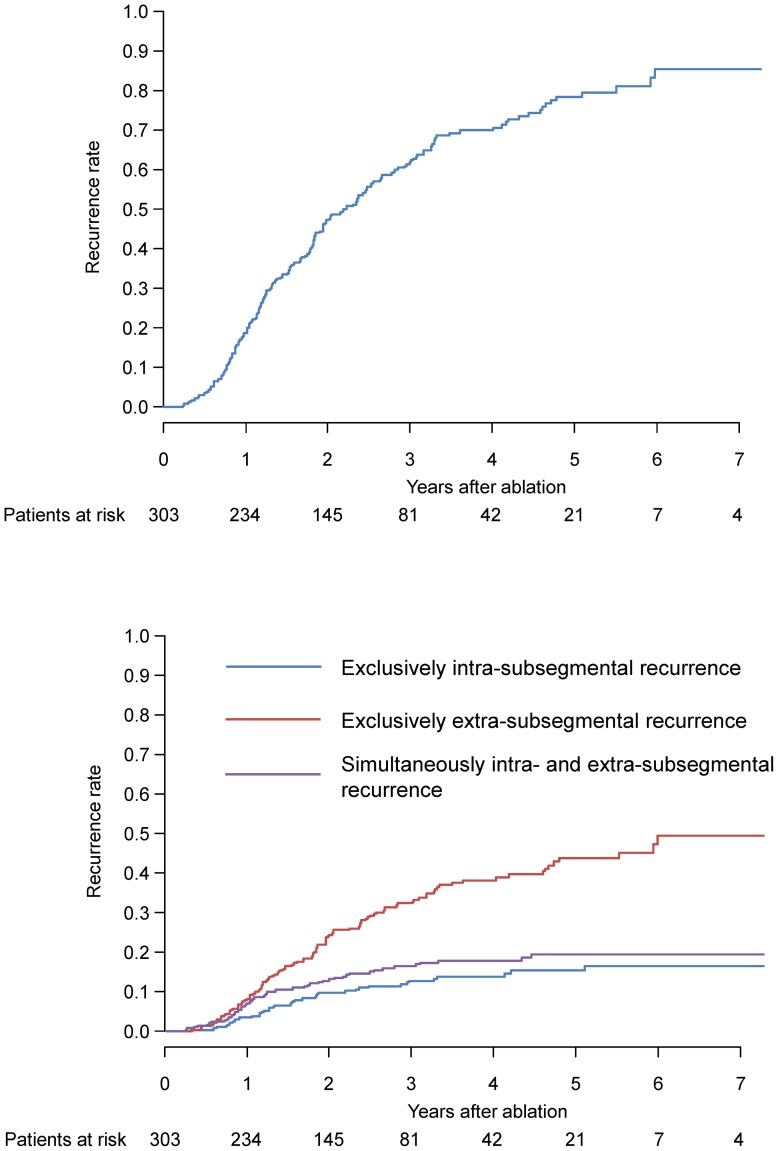
**Figure 2A:** Overall recurrence. **Figure 2B:** Recurrence rates of according to the mode of recurrence.

**Figure 3:**
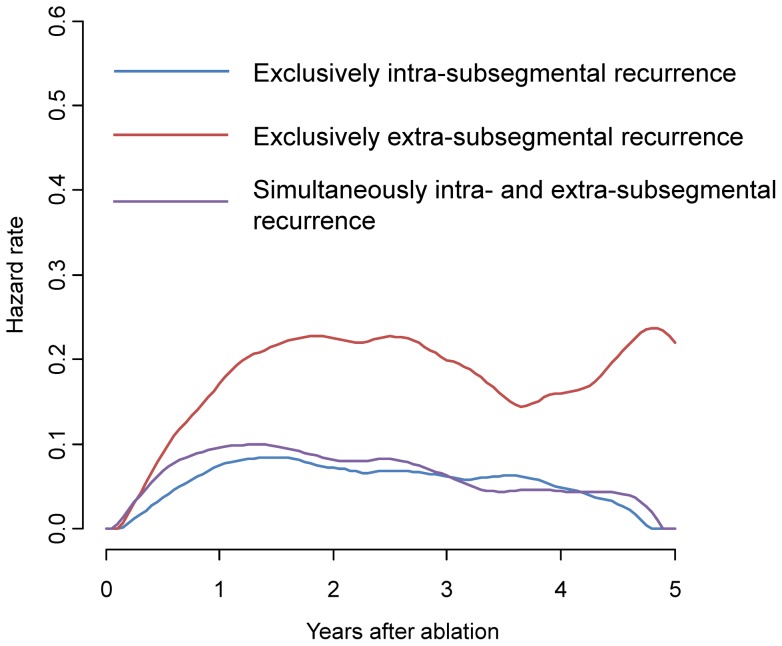
The estimated hazard function over time according to the mode of recurrence.

During the follow-up period (mean, 2.3 years; range 0.2 to 7.3 years), tumor recurrence in the HCC patient cohort was identified in 201 cases. The recurrent nodules were distributed in a total of 340 subsegments. Recurrent nodules were exclusively intra-subsegmetal in 40 patients (20%), and exclusively extra-subsegmental in 110 patients (55%, Fig. 1). Simultaneous intra- and extra-subsegmental recurrence was observed in the remaining 51 patients (25%). The diagnosis of recurrence revealed that 104, 39, 22, 17 and 19 patients had 1, 2, 3, 4–5, and >5 tumors, respectively. Local tumor progression was identified in 10 patients, among which two individuals had simultaneous extra-subsegmental recurrent nodules. Two patients with extrahepatic recurrence (one lymph node and one left adrenal grand) were categorized as extra-subsegmental. Neoplastic seeding, which was categorized as intra-subsegmental recurrence, was observed as the first recurrence in two patients. Details of the distribution of original and recurrent nodules based on subsegments are listed in Table 2. The observed proportion of recurrent nodules in the same subsegment as the original nodule was 0.268, whereas the expected probability that the subsegments of original and recurrent nodules were the same, assuming a random distribution, was 0.154. The kappa coefficient was calculated as 0.135 (95% CI, 0.079–0.190; P<0.001). When patients with a local tumor progression or neoplastic seeding were excluded from this calculation, the kappa statistic decreased to 0.101 (95% CI, 0.046–0.156; P<0.001). The cumulative rates of overall recurrence at 1, 3, and 5 years were 19.6%, 61.8%, and 78.3%, respectively (Fig. 2A). Cumulative rates of exclusively intra-subsegmental, exclusively extrasubsegmental and simultaneously intra- and extra-subsegmental recurrence were 3.4%, 8.1%, and 7.1% at 1 year, 12.7%, 32.7%, and 16.4% at 3 years, and 15.3%, 43.6%, and 19.4% at 5 years, respectively (Fig. 2B). The estimated hazard function curves according to the three types of recurrence showed a similar pattern over the first 4 years. Then only the hazard rate of exclusively extra-subsegmental recurrence increased whereas the hazard rate of the other two types of recurrence decreased (Fig. 3).

**Table 2:** Distribution of the Original and Recurrent Tumors Divided by Subsegment.

	Subsegment of recurrent tumor, n								
Subsegment of original tumor, n	S1	S2	S3	S4	S5	S6	S7	S8	sum
S1	2		2	1			1	2	8
S2	1		1		4	1	1		8
S3		7	10	8	3	3	4	12	47
S4		8	7	11	3	3	6	6	44
S5		2	2	5	10	2	4	7	32
S6	2	1		4	4	10	5	5	31
S7	3	8	11	9	9	9	15	7	71
S8	4	9	9	10	6	13	15	33	99
sum	12	35	42	48	39	41	51	72	340

### Risk factors related to intra- and extra-subsegmental recurrence

Univariate Cox proportional regressions revealed that the following factors were significantly associated with intra-subsegmental recurrence: tumor size, AFP, DCP, AFP-L3, platelet count and anti-HCV antibody positivity. The final model for predicting intra-subsegmental recurrence with stepwise variable selection included tumor size, AFP, platelet count and anti-HCV antibody positivity (Table 3). Factors related to extra-subsegmental recurrence that were found to be significant by univariate Cox proportional hazard regression were age, platelet count, tumor size, AFP and AFP-L3. Multivariate analysis with step-wise variable selection showed that the risk factors for extra-subsegmental recurrence were age, platelet count, tumor size, and AFP (Table 4).

**Table 3:** Univariate and Multivariate Analysis of Intrasubsegmental Recurrences (n = 303).

	Univariate		Multivariate	
Variable	HR (95% CI)	*P*	HR (95% CI)	*P*
Age per year	1.00 (0.97–1.02)	0.77		
Male gender	1.05 (0.68–1.63)	0.82		
HBsAg, positive	0.64 (0.30–1.40)	0.27		
anti-HCVAb, positive	2.08 (1.13–3.84)	0.02	2.04 (1.09–3.81)	0.03
Platelet count, ×10^4^/μL	0.95 (0.91–0.99)	0.009	0.97 (0.93–1.01)	0.09
ALT >80 IU/L	0.99 (0.58–1.71)	0.98		
Size per 1 cm	1.29 (1.08–1.55)	0.006	1.28 (1.06–1.54)	0.009
log(AFP)	1.93 (1.53–2.45)	<0.001	1.29 (1.16–1.44)	<0.001
log(DCP)	1.66 (1.18–2.33)	0.003		
AFP-L3>15%	2.02 (1.20–3.41)	0.009		

HR, hazard ratio; CI, confidence interval; HBsAg, hepatitis B surface antigen; Anti-HCVAb, anti-hepatitis C virus antibody; ALT, alanine aminotransferase, AFP, alpha-fetoprotein; DCP, des-gamma-carboxyprothrombin; AFP-L3, lens culinaris agglutinin-reactive fraction of AFP.

**Table 4:** Univariate and Multivariate Analysis of Extrasubsegmental Recurrences (n  =  303).

	Univariate		Multivariate	
Variable	HR (95% CI)	*P*	HR (95% CI)	*P*
Age per year	1.02 (1.00–1.04)	0.03	1.03 (1.01–1.05)	0.001
Male gender	1.13 (0.83–1.56)	0.44		
HBsAg, positive	0.91 (0.30–1.40)	0.69		
anti-HCVAb, positive	1.49 (1.00–2.20)	0.049		
Platelet count, ×10^4^/μL	0.94 (0.91–0.97)	<0.001	0.94 (0.92–0.97)	<0.001
ALT >80 IU/L	1.05 (0.72–1.56)	0.78		
Size per 1 cm	1.32 (1.16–1.51)	<0.001	1.39 (1.21–1.60)	<0.001
log(AFP)	1.53 (1.27–1.85)	<0.001	1.37 (1.12–1.68)	0.03
log(DCP)	1.29 (0.96–1.73)	0.1		
AFP-L3>15%	1.66 (1.09–2.52)	0.018		

HR, hazard ratio; CI, confidence interval; HBsAg, hepatitis B surface antigen; Anti-HCVAb, anti-hepatitis C virus antibody; AST, aspartate aminotransferase: ALT, alanine aminotransferase, AFP, alpha-fetoprotein; DCP, des-gamma-carboxy prothrombin; AFP-L3, lens culinaris agglutinin-reactive fraction of AFP.

### Treatment of recurrent HCC and associated survival outcomes

**Figure 4:**
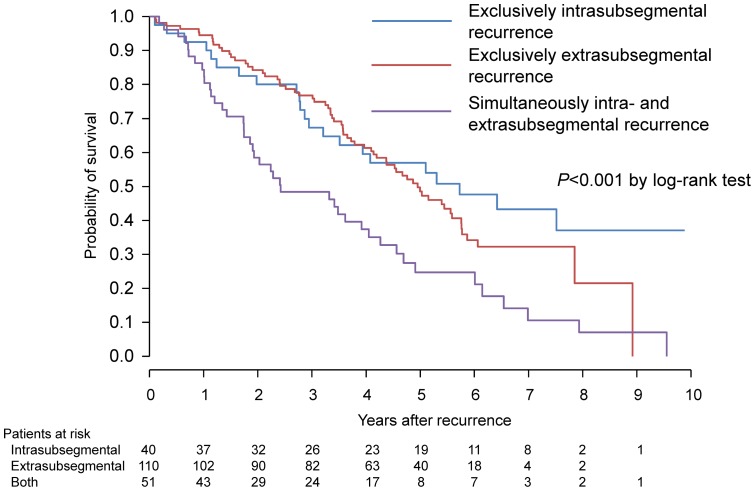
Cumulative survival probability after the diagnosis of recurrence according to the mode of recurrence.

Among the 40, 110 and 51 patients in whom recurrent HCC was found to be exclusively intra-subsegmental, exclusively extra-subsegmental, and simultaneously intra- and extra-subsegmental, 37 (92.5%), 99 (90.8%) and 42 (82.3%), respectively, were treated using RFA. Of the three patients with an exclusively intra-segmental recurrence, one individual was treated by hepatic resection and one patient was treated by TACE. The remaining patient received best supportive care because of deterioration in liver function. During the follow up period up to December 31, 2010, 130 patients died and 9 patients were lost to follow-up. The median survival time (95% CI) was 5.72 (3.51-NA) years in patients with exclusively intra-subsegmental recurrence, 4.95 (4.19–5.76) years in patients with exclusively extra-subsegmental recurrence, and 2.43 (1.90–4.26) years n patients with simultaneously intra- and extra-subsegmental recurrence, respectively (P<0.001 by log-rank test, Fig. 4). Univariate Cox regression analysis revealed that patients with simultaneously intra- and extra-subsegmental recurrences had a significantly poorer survival than those with an exclusively intra-subsegmental recurrence (hazard ratio, 2.39; 95% CI, 1.32–4.02; P = 0.001), wheareas this difference became non-significant (HR, 1.91; 95% CI, 0.96–3.80; P = 0.07) when adjusted using other significant factors in univariate analysis (Table 5). No differences in the survival outcomes between patients with exclusively intra- and extra-subsegmental recurrences were observed by univariate and multivariate analysis. Finally overall survival rates after the initial RFA at 1, 3, 5, 7 and 10 years were 96.7%, 81.4%, 62.4%, 49.0%, and 31.1%, respectively.

**Table 5:** Univariate and Multivariate Analysis of Survival after Recurrence*.

	Univariate		Multivariate	
Variable	HR (95% CI)	*P*	HR (95% CI)	*P*
Exclusively extra-subsegmental recurrence vs. exclusively intra-subsegmental recurrence	1.18 (0.73–1.92)	0.50	1.28 (0.76–2.16)	0.35
Simultaneously intra- and extra-subsegmental recurrence vs. exclusively intra-subsegmental recurrence	2.39 (1.32–4.02)	0.001	1.91 (0.96–3.80)	0.07
Age, per 1year	1.02 (1.00–1.05)	0.04	1.04 (1.02–1.07)	0.001
Male gender	1.06 (0.75–1.52)	0.73		
HBsAg, positive	0.74 (0.40–1.38)	0.34		
anti-HCVAb, positive	1.24 (0.77–1.99)	0.39		
Child-Pugh Score, per 1 point	1.45 (1.28–1.63)	<0.001	1.44 (1.27–1.63)	<0.001
Platelet count, per 10^4^/μL	0.98 (0.95–1.01)	0.15		
ALT >80 IU/L	0.81 (0.49–1.33)	0.40		
Size >2cm	1.57 (1.11–2.23)	0.01	1.54 (1.06–2.23)	0.02
Multinodular	1.66 (1.18–2.35)	0.004	1.02 (0.63–1.66)	0.92
log(AFP)	1.45 (1.20–1.85)	<0.001	1.13 (1.01–1.26)	0.04
log(DCP)	1.61 (1.21–2.15)	0.001	1.21 (1.06–1.38)	0.004
AFP-L3 >15%	1.93 (1.25–2.98)	0.003	1.17 (0.70–1.96)	0.56

* Clinical data at the diagnosis of recurrence were adopted.

HR, hazard ratio; CI, confidence interval; HBsAg, hepatitis B surface antigen; Anti-HCVAb, anti-hepatitis C virus antibody; AST, aspartate aminotransferase: ALT, alanine aminotransferase, AFP, alpha-fetoprotein; DCP, des-gamma-carboxy prothrombin; AFP-L3, lens culinaris agglutinin-reactive fraction of AFP.

## Discussion

Recurrences of HCC are more complicated than those of other solid tumors as they can arise in two distinct forms: de novo carcinogenesis and intrahepatic metastasis [32]. Systematic subsegmentectomy may be effective in treating such patients if the distribution of the hematogenous spread of cancer cells correlates with the physical distance from the original tumor or local portal venous flow. Indeed, in the present study we showed from our data that the location of recurrent nodules was weakly but significantly related to that of the original tumor, even after the exclusion of local tumor progression from the analysis. Given that exclusively intrasubsegmental recurrence in this study could be prevented by subsegmentectomy, through a simple calculation, one fifth of patients who received locally curative RFA might have benefitted if they had received systematic subsegmentectomy. However, it should be mentioned in this regard that those patients who had avoided an intra-subsegmental recurrence owing to a systematic subsegmentectomy would have subsequently encountered tumor recurrence in the remnant liver, and the actual risk reduction of recurrence would therefore be smaller. Actually recurrence-free survival at 10 years after systematic subsegmentectomy was reported to be only 9.4% in a previous nation-wide survey [33].

The risk factors related to de novo carcinogenesis and hematogenous intrahepatic metastasis would be expected to be different. The factors responsible for HCC development, such as fibrosis stage, age, gender, and presence of viral hepatitis, may also affect de novo carcinogenesis [34], [35]. On the other hand, factors related to the primary tumor, such as the size and number of tumor nodules, pathological grade(s), the presence of vascular invasion, and positivity of tumor markers, may affect the possibility of intrahepatic occult metastasis at the time of initial treatment. We speculated that there would be differences between the risk factors for intra- and extrasubsegmental recurrence since the former would more strongly correlate with hematogenous intrahepatic metastasis. However the risk factors related to intra- and extrasubsegmental recurrence were found to be quite similar except that old age was a risk factor for only extrasubsegmental recurrence.

Previous reports suggested the hazard function of de novo carcinogenesis and hematogenous intrahepatic metastasis would be different [36], [37]. The hazard function of the former is assumed to be gradually increasing over time whereas that of the latter has a peek within two years. And the actual hazard function represents the sum of the two curves. The estimated hazard function of exclusively extra-subsegmental recurrence in this study seemed compatible with the previous reports. However we should be careful to interpret the results because the number at risk at year 4 or 5 was limited.

A previous large scale cohort study of the prognosis of patients with HCC treated by liver transplantation has reported that microvascular invasion is the most important predictor of a poor outcome [38]. This suggests that even if the whole liver is removed, there may be remaining circulating tumor cells that have resulted from tumor nodule invasion of the microvessels. It has also been reported that microsatellite metastatic nodules surrounding the main tumor are associated with microvascular invasion and indicate a higher risk of tumor recurrence after liver transplantation [39]. Hence, the impact of removing a tumor-bearing subsegment, including microvascular invasions or microsatellite nodules, which is thought to be a major advantage of resection over RFA, might be more limited than previously considered.

In this study factors that were supposed to be related to de novo carcinogenesis (e.g., lower platelet count and HCV infection) were risk factors for intra-subsegmental recurrence as well as extra-subsegmental recurrence. The risk of recurrence due to de novo carcinogenesis might be reduced by a subsegmentectomy according to the resected liver volume. However, as most patients with HCC have chronic liver disease, removal of non-cancerous liver parenchyma may have a negative impact on long-term survival, especially for those individuals with impaired liver regeneration. Therefore, the key issue is to what extent the liver parenchyma should be removed to sufficiently treat the patient on a case by case basis. It may be speculated that extensive resection could be tolerable and beneficial to those who have a well-preserved capacity for liver regeneration [40].

There is no doubt that tumor recurrence deteriorates the long-term prognosis for HCC patients. However it is also true that there are effective, sometimes potentially curative treatments for recurrent HCC. The re-resection after recurrence of HCC is indicated in 10–30% of patients [41]–[43] and percutaneous ablation can be repeatedly performed [20], [44], [45]. Indeed, 37 of 40 patients analyzed in this study who had recurrent nodules confined to the same subsegment as the original tumor were successfully re-treated with RFA. No differences in the survival outcomes were observed between patients with solely intra- or extra-subsegmental recurrences. Hence, the impact of the first recurrence on overall survival may be smaller for HCC compared with other gastrointestinal malignancies such as stomach cancer or colorectal cancer.

We would touch upon the possibility that TACE prior RFA might affect the results. Approximately one third of patients underwent TACE prior RFA. TACE prior RFA can increase the ablated area through the blockade of arterial flow and may decrease the risk of local tumor progression. However the influence would be minimal because local tumor progression was found in only 10 patients and the location of recurrent nodules was weakly but significantly related to that of the original tumor, even after the exclusion of local tumor progression.

In conclusion, the vast majority of recurrent nodules in HCC patients were found to be independent of the subsegment of the original tumor. In addition, whether these recurrences were intra-subsegmental or extrasubsegmental had no impact on the survival outcomes.
